# Conformational Ensembles of α-Synuclein Derived Peptide with Different Osmolytes from Temperature Replica Exchange Sampling

**DOI:** 10.3389/fnins.2017.00684

**Published:** 2017-12-07

**Authors:** Salma Jamal, Anchala Kumari, Aditi Singh, Sukriti Goyal, Abhinav Grover

**Affiliations:** ^1^Department of Bioscience and Biotechnology, Banasthali University, Tonk, India; ^2^Department of Biotechnology, TERI School of Advanced Studies, New Delhi, India; ^3^School of Biotechnology, Jawaharlal Nehru University, New Delhi, India

**Keywords:** Parkinson's disease, intrinsically disordered protein, α-synuclein, osmolytes, replica exchange molecular dynamics

## Abstract

Intrinsically disordered proteins (IDP) are a class of proteins that do not have a stable three-dimensional structure and can adopt a range of conformations playing various vital functional role. Alpha-synuclein is one such IDP which can aggregate into toxic protofibrils and has been associated largely with Parkinson's disease (PD) along with other neurodegenerative diseases. Osmolytes are small organic compounds that can alter the environment around the proteins by acting as denaturants or protectants for the proteins. In the present study, we have conducted a series of replica exchange molecular dynamics simulations to explore the role of osmolytes, urea which is a denaturant and TMAO (trimethylamine N-oxide), a protecting osmolyte, in aggregation and conformations of the synuclein peptide. We observed that both the osmolytes have significantly distinct impacts on the peptide and led to transitions of the conformations of the peptide from one state to other. Our findings highlighted that urea attenuated peptide aggregation and resulted in the formation of extended peptide structures whereas TMAO led to compact and folded forms of the peptide.

## Introduction

Parkinson's disease (PD) is the second most common degenerative disorder of the central nervous system after Alzheimer's disease (AD). The disease affects approximately seven million people worldwide and around one million people in the United States (Yao et al., 2013). PD is most common in case of elderly population, around 60 years, and the rate rises from 1% in those above 60 years to 4% in the population of 80% (Sampaio et al., 2002). A total of about 103,000 deaths were reported globally in 2013 from PD increasing from 44,000 deaths in 1990 (GBD 2013 Mortality Causes of Death Collaborators, 2015). PD is a chronic and progressive disorder with the symptoms worsening over time. The most common symptoms include tremor of hands, arms, jaw, face, and legs, bradykinesia or slowness of movement, rigidity or stiffness and resistance of the limbs movement, postural instability which leads to impaired balance and frequent falls (Jankovic, 2008).

In terms of pathophysiology, PD has been linked to α-synuclein due to the aberrant accumulation of the protein in the brain in the form of Lewy bodies (Galpern and Lang, 2006). Multiple ways have been reported in which α-synuclein can lead to PD pathogenesis, however the most common mechanism states the abnormal aggregation of the protein/peptide in soluble oligomeric forms termed as protofibrils, which are toxic and lead to disruption of homeostasis and neuronal cell death (Emmanouilidou et al., 2010). α-synuclein has been involved in other neurodegenerative diseases other than PD termed as synucleinopathies which include dementia with Lewy bodies, and multiple system atrophy, Down's syndrome and the Lewy body variant of AD (McCann et al., 2014).

Human α-synuclein is a 140 amino acid residue protein found in high concentrations at presynaptic terminals in the brain and predominantly expressed in substantia nigra, hippocampus, thalamus, and cerebellum (Gallea and Celej, 2014). Alpha-synuclein has three distinct regions which include an amphipathic N-terminal domain (residues 1–60), a central hydrophobic region, the non-amyloid-beta component (NAC) (residues 61–95) and a highly acidic C-terminal domain (residues 96–140). Synucleins have usually been described as intrinsically disordered proteins (IDP) as they lack a stable three-dimensional structure (van Rooijen et al., 2009). The completely random and unfolded structures of IDP have been known to promote aggregated protein conformations subsequently leading to various diseases (Gregersen et al., 2005). Since the monomeric form of α-synuclein has been shown to aggregate into fibrils and plays a role in PD pathogenesis, structural analysis of the monomeric form of the α-synuclein peptide to understand the conformational changes which result in its aggregated state is very crucial.

Proteins are slightly stable and the conformational state of the proteins can easily be altered by change in the thermodynamic environment of the proteins and through addition of solvents (Prabhu and Sharp, 2006), small molecule cosolvents, crowding agents (Zhou et al., 2008), and osmolytes (Harries and Rösgen, 2008). Osmolytes are naturally occurring small compounds commonly referred to as chemical chaperones which may either cause a protein to fold into native stable conformation as protectant osmolytes [that include trimethylamine N-oxide (TMAO)] or may result in an unfolded ensemble termed as denaturants (that include urea; Kumar, 2009). Urea is an organic compound with a chemical formula, CO(NH_2_)_2_ which is used as a powerful denaturant (Gordon and Jencks, 1963) while TMAO is a small molecule with chemical formula, ((CH_3_)_3_NO) which is a protein stabilizer (Zou et al., 2002). Various studies have been performed to compare and explore the conformations of peptides in the presence of urea and TMAO. Kokubo et al. (2011) investigated the nature of protecting osmolyte, TMAO, and denaturing osmolyte, urea, using standard Molecular dynamics (MD) simulations and free energy analysis on a deca-alanine peptide model. Their analysis reported that the difference in the behavior of peptide in two different osmolytes solutions was due to the van der Waals (vdW) interactions and electrostatic interactions (Kokubo et al., 2011). Mondal et al. (2013) also used MD simulations and showed that 1M TMAO and 7M urea solutions acted in a significantly different manner on the polymer chains. Their study showed that while TMAO suppressed the formation of extended conformations of the polymer chain, urea promoted the formation of extended conformations (Mondal et al., 2013).

MD simulations studies have been widely performed to investigate the structural aspects of the IDP proteins such as amyloid-β protein (Aβ), α-synuclein, tau protein, and human Amylin, etc. Lockhart et al. (2012) used an all-atom explicit solvent replica exchange molecular dynamics (REMD) and studied the interactions between monomeric form of amyloid-beta and ibuprofen and reported that ibuprofen binding to the peptide was dominated by hydrophobic effect and that the conformational ensemble of Aβ is mainly determined by the formation of Asp23-Lys28 salt bridge and the hydrophobic interactions (Lockhart et al., 2012). Eugene et al. (2014) studied the characterization of α-synuclein oligomers from monomer to dimer and further to trimer focusing on the 35-residue NAC fragment using REMD (Eugene et al., 2014). Levine et al. (2015) explored the role of osmolytes, urea, and TMAO, in regulating the conformations of tau protein and reported that the urea and TMAO resulted in population shifts in case of monomeric forms (Levine et al., 2015). Mo et al. (2016) performed extensive REMD on human islet amyloid polypeptide (hiAPP) in the presence of epigallocatechin gallate (EGCG) to study the molecular mechanism by which ECGC inhibits hiAPP aggregation (Mo et al., 2016).

Although a number of studies have been performed to explore the role of osmolytes in conformational behavior of proteins, the mechanistic details of osmolyte-mediated compaction/unfolding in case of the intrinsically disordered protein, α-synuclein, which leads to PD still need further investigation. Various studies have shown the conformational changes in the monomeric α-synuclein consequently leading to its fibrillary aggregation responsible for PD (Spillantini et al., 1997; Moore et al., 2005) and thus depicting and understanding the conformations of monomeric form of α-synuclein is essential. In the present study, we have explored the effects of urea and TMAO in regulating the conformational properties focusing on the structural aspect of α-synuclein peptide, which is intrinsically disordered in its monomeric form. We used water as explicit solvent model and carried out REMD simulations for a period of 100 ns per replica on a small segment of α-synuclein, TGVTAVA. This segment has been reported to be an amyloid forming segment, residues 72–78, which fall into NAC region of the protein which is responsible for α-synuclein aggregation and its conversion into oligomers and resultant toxic fibrils found in PD-inflicted brains (Li et al., 2014). The synuclein peptide region investigated in the present study, residues 72–78, falls in the NAC core, GAVVTGVTAVA residues 68–78, as well as subNACore, AVVTGVTAV residues 69–77, described by Rodriguez et al. (2015). We observed remarkably different effects of the denaturant osmolyte urea and protectant osmolyte TMAO on the peptide fragment. Urea enhanced the formation of extended structures while TMAO resulted in the formation of compact and folded conformations. The results provided in the present study will provide a detailed understanding of the effects of different osmolytes on the behavior of the segment of the intrinsically disordered α-synuclein protein.

## Methodology

### Initial structure of the peptide

The crystal structure of the amyloid forming segment from α-synuclein (residues 72–78) with a sequence, TGVTAVA (Li et al., 2014), was obtained in monomeric form from Protein Data Bank (Berman et al., 2000), PDB code: 4R0U. The peptide was prepared using Protein Preparation Wizard available from Schrodinger (Sastry et al., 2013) during which the N- and C-termini of the peptide were capped. The peptide used in the present study was in fully extended conformation.

### Replica exchange molecular dynamics (REMD) simulations

The GROMACS 4.5.7 (Van Der Spoel et al., 2005) software package was used to perform the simulations with GROMOS 43a1 (van Gunsteren et al., 1996) force field which has been used earlier by Cao et al. (2011). The peptide was placed in a triclinic box which was filled with single point charge (Berendsen et al., 1981) water model consisting of 3,299 water molecules and periodic boundary conditions were used. The sodium and chloride ions were added to neutralize the positive charge of the peptide. The choice of using sodium chloride is based on the large number of previous molecular dynamics studies on alpha-synuclein where sodium chloride has been used (Herrera et al., 2008; Cino et al., 2012; Tian et al., 2012; Vekrellis and Stefanis, 2012; Vermaas and Tajkhorshid, 2014; Mane and Stepanova, 2016). Long range electrostatic interactions were computed using Particle Mesh Ewald method (Darden et al., 1993; Essmann et al., 1995) with a grid spacing of 0.12. The short range electrostatic interactions in addition to Van der Waals interactions were calculated with a cut-off of 1 nm. The leap-frog integrator algorithm was used for integrating Newton's equations of motion with a time step of 2 fs. Prior to MD simulations, the energy minimization was performed using steepest descent algorithm. Initially, the systems were simulated under Isothermal-Isobaric ensemble (NPT, constant number of atoms, pressure, and temperature) at 300 K temperature and 1 bar pressure using the Berendsen weak-coupling thermostat and barostat (Berendsen et al., 1984) for 2 ns. This was followed by NVT (constant number of atoms, volume, and temperature) ensemble using a Nose–Hoover thermostat (Hoover, 1985). Linear constraint solver (Hess, 2008) algorithm was used to constraint the peptide bond lengths.

A total of 18 replicas were generated in the temperature range from 300 to 347K (T = 300.61K, 303.24K, 305.89K, 308.56K, 311.24K, 313.94K, 316.67K, 319.41K, 322.18K, 324.96K, 327.76K, 330.58K, 333.42K, 336.29K, 339.17K, 342.08K, 345.00K, 347.94K) with an average exchange probability of 25%. The temperature was predicted according to the method described by Patriksson and van der Spoel (2008). Each replica at a fixed temperature was able to explore the conformational space of the peptide. The high temperature replicas enabled the crossing of energy barriers of the system by the low temperature replicas thus facilitating the exploration of new conformational spaces by low temperature replicas and vice versa. Exchange between the replicas was attempted every 2 ps and each replica was run for 100 ns and the data was collected every 10 ps. The peptide was simulated in the presence of two osmolytes, urea and TMAO using the already developed models by Weerasinghe and Smith (2003) and Larini and Shea (2013), respectively. The concentrations of the osmolytes, 5M for UREA (662 molecules) and 2M for TMAO (65 molecules), were used in accordance with the previous studies (Kokubo et al., 2011).

### Measures for molecular dynamics analysis

MD analysis was performed using various tools available from GROMACS which include g_hbond, g_sas, g_cluster, g_polystat and g_rms. g_hbond was used to measure and analyze intra-molecular hydrogen bonds with the cut-off of 2.5 Å or smaller for oxygen-hydrogen distance. g_sas computed surface area of the peptide which was accessible to solvent using the double cubic lattice method(Eisenhaber et al., 1995). g_rms was used to check the stability of the simulation. g_polystat determined the average end-to-end distance (R_ee_) and also computed the radius of gyration (R_g_) as a function of time which is the measure of compactness of a protein. The end-to-end distance was calculated from the acetylated cap's center of mass, C-termini to the amidated cap's center of mass, N-termini. g_cluster clustered the structures using algorithm as described in Daura et al. (2001) which uses a cut-off to count the neighbors based on root mean square deviation and puts a structure with largest number of neighbors in one cluster along with its neighbors (Daura et al., 2001). The cut-off for used in the present study for cluster analysis was 0.25 nm.

## Results

**Figure 1:**
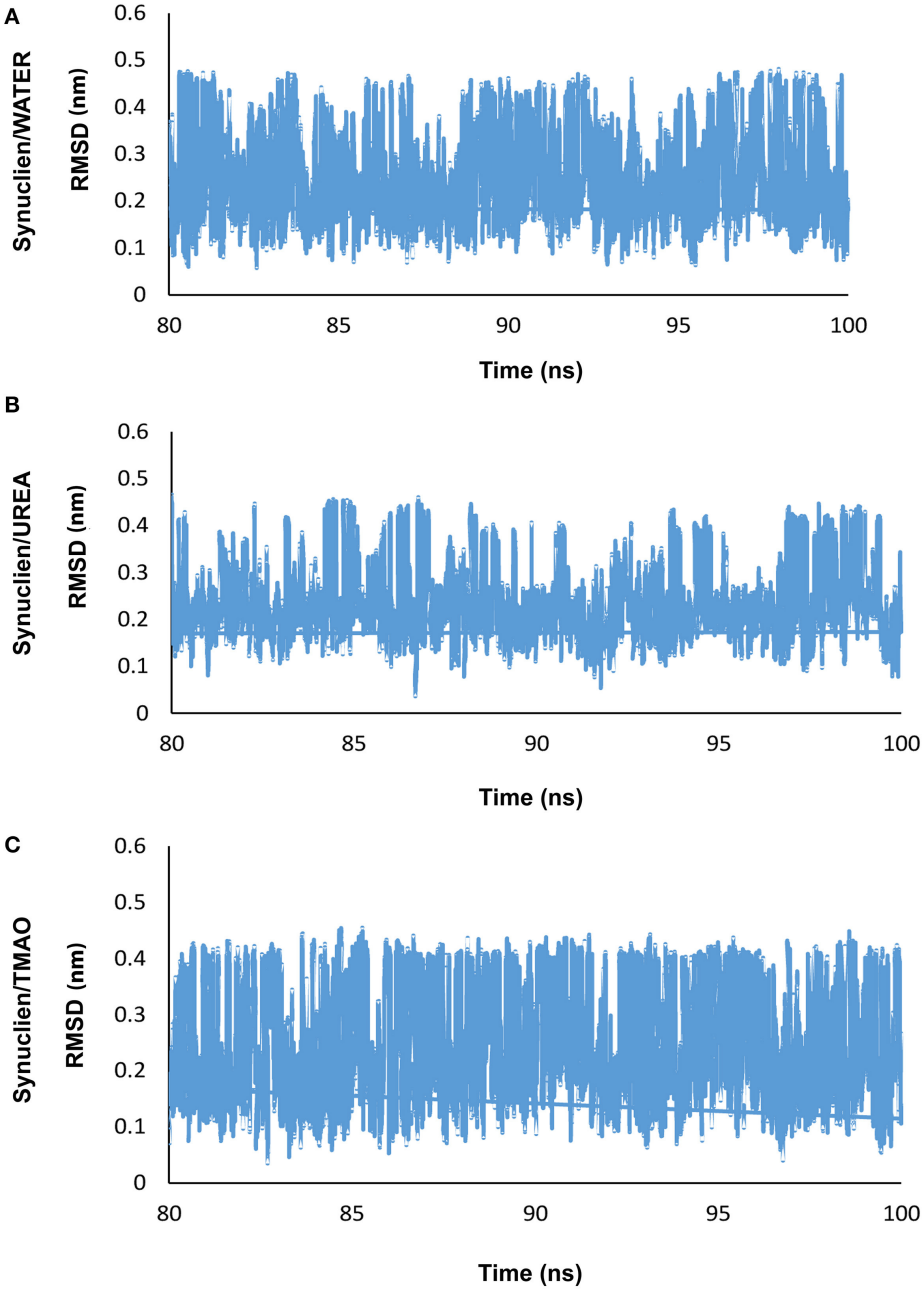
Shows the RMSD plots for **(A)** water (Synuclein_water_) and two osmolytes, **(B)** UREA (Synuclein_Urea+water_), and **(C)** TMAO (Synuclein_TMAO+water_) which demonstrates that the peptide adopted various conformations.

In the present study, REMD was performed on α-synuclein protein segment, TGVTAVA, in the presence of water (Synuclein_water_) and two osmolytes, UREA (Synuclein_Urea+water_) and TMAO (Synuclein_TMAO+water_) to study conformational sampling of the peptide. A total of 18 replicas in the temperature range, 300–350 K, were generated and REMD simulation analyses was carried out for the replica at 300 K temperature. Root mean square deviation (RMSD) was calculated for all the three systems, Synuclein_water_, Synuclein_Urea+water_, and Synuclein_TMAO+water_. As can be seen in Figure 1, the peptide adopted multiple conformations as is indicated by fluctuation of RMSD values for water (Synuclein_water_) and two osmolytes, UREA (Synuclein_Urea+water_) and TMAO (Synuclein_TMAO+water_) systems.

### Contrasting effects of urea and TMAO on α-synuclein

**Figure 2:**
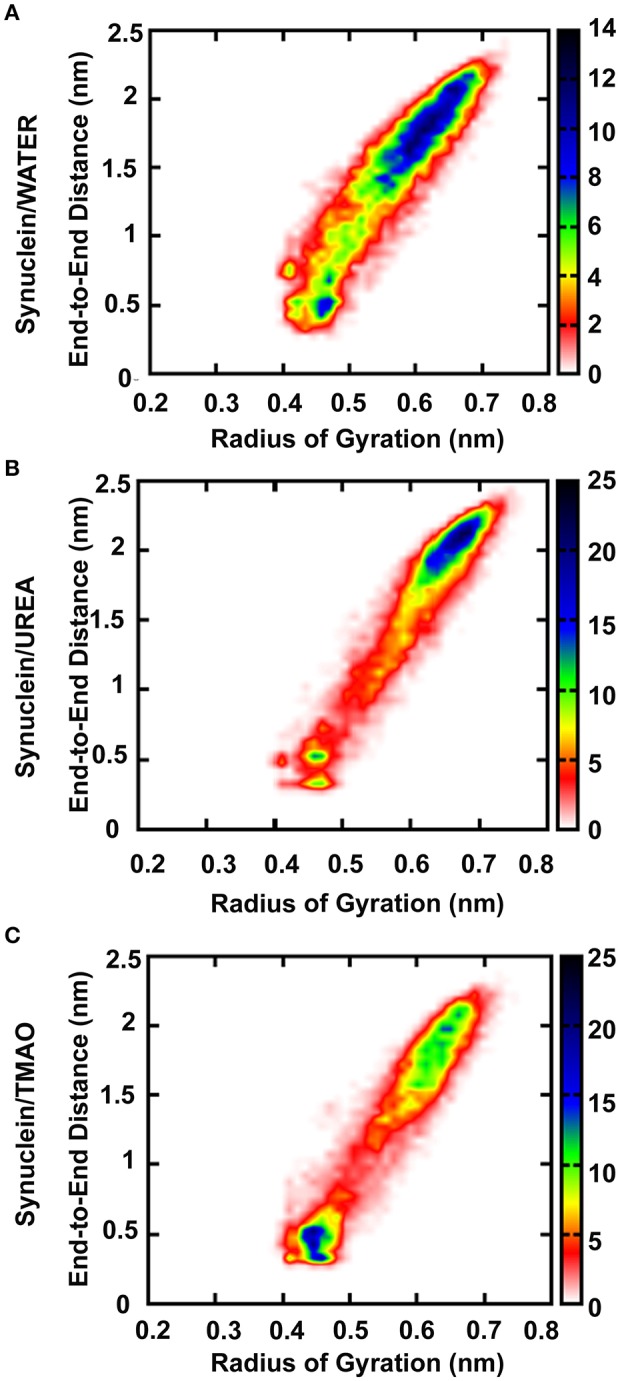
Population densities of Synuclein peptide conformations **(A)** Synuclein (water), **(B)** Synuclein (urea), and **(C)** Synuclein (TMAO), R_ee_ i.e., peptide end-to-end distance (C to N terminal) and R_g_ i.e., radius of gyration about its center of mass. The densely populated region has been shown in blue color while the other less populated regions have been shown in green, yellow, red, and white color.

Osmolytes are the compounds that protect cells against osmotic pressure. However, they can have substantial effects on the stability of the protein. In the present study, we have shown that the protectant 2M TMAO and denaturant 5M Urea osmolytes show remarkably different impacts on the peptide conformation. Addition of urea promotes the formation of more extended structures for Synuclein_Urea+water_ peptide while TMAO, Synuclein_TMAO+water_ suppresses the formation of extended conformations resulting in the compact and folded forms of the peptide. In case of water, the peptide was in extended state and addition of TMAO led to formation of compact forms of the peptide as is evident from Figure 2. Since the peptide used in the present study was already in extended state, there were not much differences in case of urea as it promotes unfolding and hence the peptide remained in extended state in Synuclein_Urea+water_. However, we obtained a folded and compact conformation of the peptide in TMAO containing system. We have generated population densities plot as a function of R_ee_ and R_g_ for all the three systems, Synuclein_water_, Synuclein_Urea+water_, and Synuclein_TMAO+water_ (Figures 2A–C). The densely populated region has been shown in blue while the other less populated regions have been shown in green, yellow, red, and white. From the population density plot, it was observed that extended structures were more populated in case of urea. On the contrary there are fewer extended conformations with TMAO than with urea. Table 1 shows the values for R_ee_ and R_g_ for all the three systems, Synuclein_water_, Synuclein_Urea+water_, and Synuclein_TMAO+water_. Maximum average values, 1.53 nm R_ee_ and 0.59 nm R_g_, were observed in case of Synuclein_Urea+water_ indicating toward the unfolded form of the peptide in the presence of urea. In case of TMAO, the values for R_ee_ (1.27 nm) and R_g_ (0.54 nm), were less as compared to water which shows that the peptide got further compact and folded in the presence of TMAO. In case of Synuclein_water_ system, the values for R_ee_ and R_g_ were 1.39 and 0.55 nm, respectively. Similar results have been obtained for compact/extended structures in protectant/denaturing osmolytes in case of α-synuclein using the single-molecule fluorescence resonance energy transfer method (Ferreon et al., 2012).

**Table 1:** Average R_ee_ i.e., peptide end-to-end distance (C to N terminal) and R_g_ i.e., radius of gyration of Synuclein monomer.

**Synuclein peptide in respective solvent/s**	**Average R_ee_ (nm)**	**Average R_g_ (nm)**
Synuclein_water_	1.39	0.55
Synuclein_Urea+water_	1.53	0.59
Synuclein_TMAO+water_	1.27	0.54

Additionally, we also analyzed the effects of osmolytes on the α-synuclein peptide at higher temperatures, (T = 311.24K, 322.18K, 333.42K, 342.08K, and 347.94K) and observed that the peptide was in folded state in the presence of urea at all the temperatures. However, it was also observed that the peptide, which was in folded form at 300K, no longer remained in folded form at higher temperatures, 333.42K, 342.08K, and 347.94K, in the presence of TMAO and that the peptide adopted extended conformations. The population density plots of the peptide monomer for all the three systems, Synuclein_water_, Synuclein_Urea+water_, and Synuclein_TMAO+water_, at various temperatures have been provided as Figures S1–S5. Moreover, we also computed the average Ree and Rg for all the three systems, Synuclein_water_, Synuclein_Urea+water_, and Synuclein_TMAO+water_ (Table S1). The average values for Ree and Rg in case of Synuclein_Urea+water_ system were greater in comparison to values for Synuclein_TMAO+water_ system which indicates that the peptide was enriched in extended conformations in the presence of urea while folded states were prevalent in case of TMAO-containing system. However, at higher temperatures, extended conformations of the peptide were observed in the presence of TMAO, but to a lesser extent in comparison to urea.

**Figure 3:**
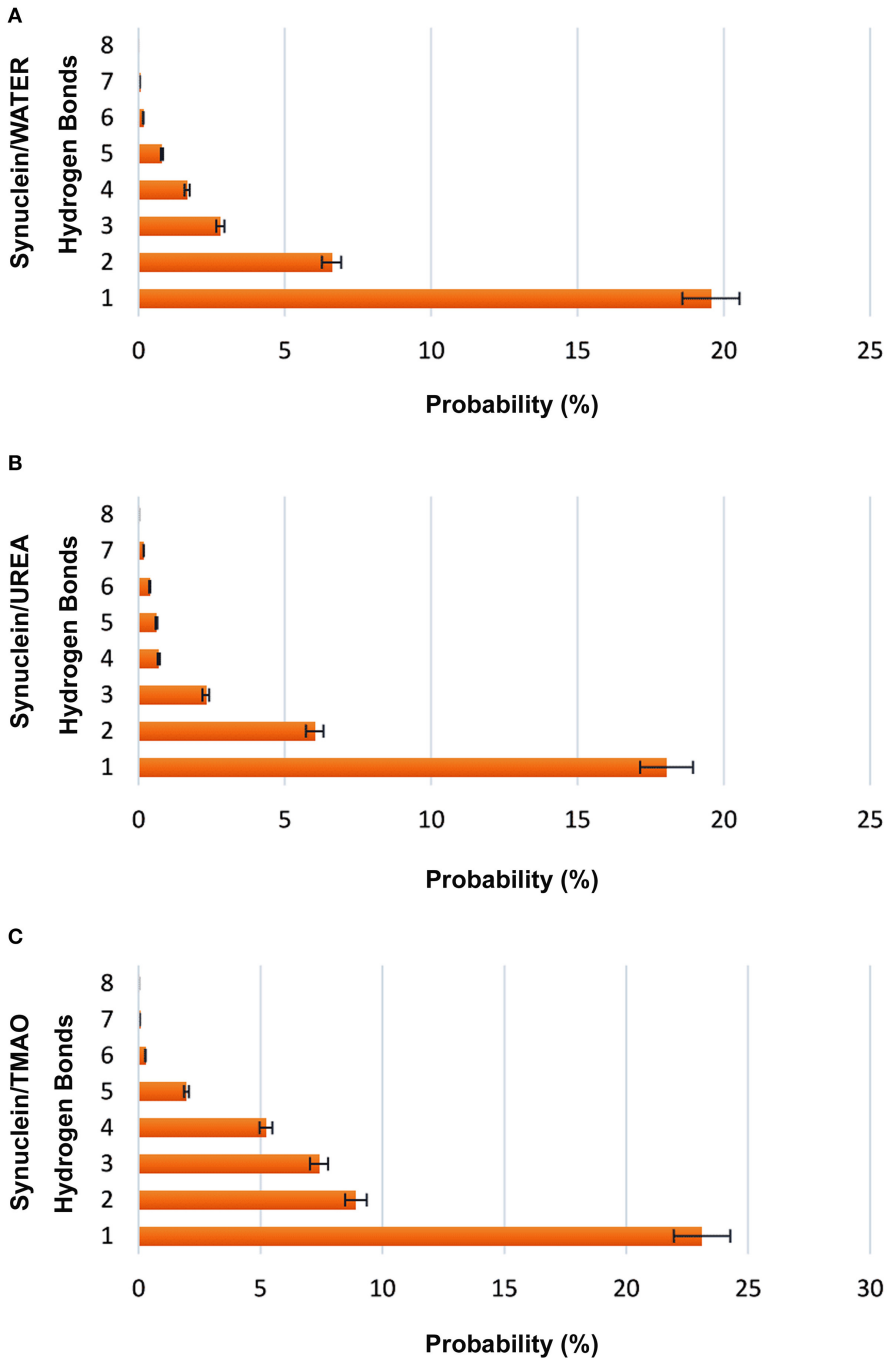
Probabilities of forming intramolecular hydrogen bonds (C=O to N-H) in case of all the three systems, **(A)** Synuclein_water_, **(B)** Synuclein_Urea+water_, and **(C)** Synuclein_TMAO+water_. It is evident that the probability of hydrogen bonds formation was decreased in case of urea indicating the extended states of peptide. TMAO containing system showed higher probability in comparison to urea and water.

A significant decrease in the number of hydrogen bonds in case of urea was observed indicating the formation of extended conformations as is evident from Figure 3. In case of TMAO, 23% probability was observed for having at least one hydrogen bond which was much higher than urea and water which had a probability of 17 and 19.5%, respectively. The probability of hydrogen bond formation in case of all the three systems Synuclein_water_, Synuclein_Urea+water_, and Synuclein_TMAO+water_ is shown in Figure 3.

### Peptide hydration in urea and TMAO containing systems

**Figure 4:**
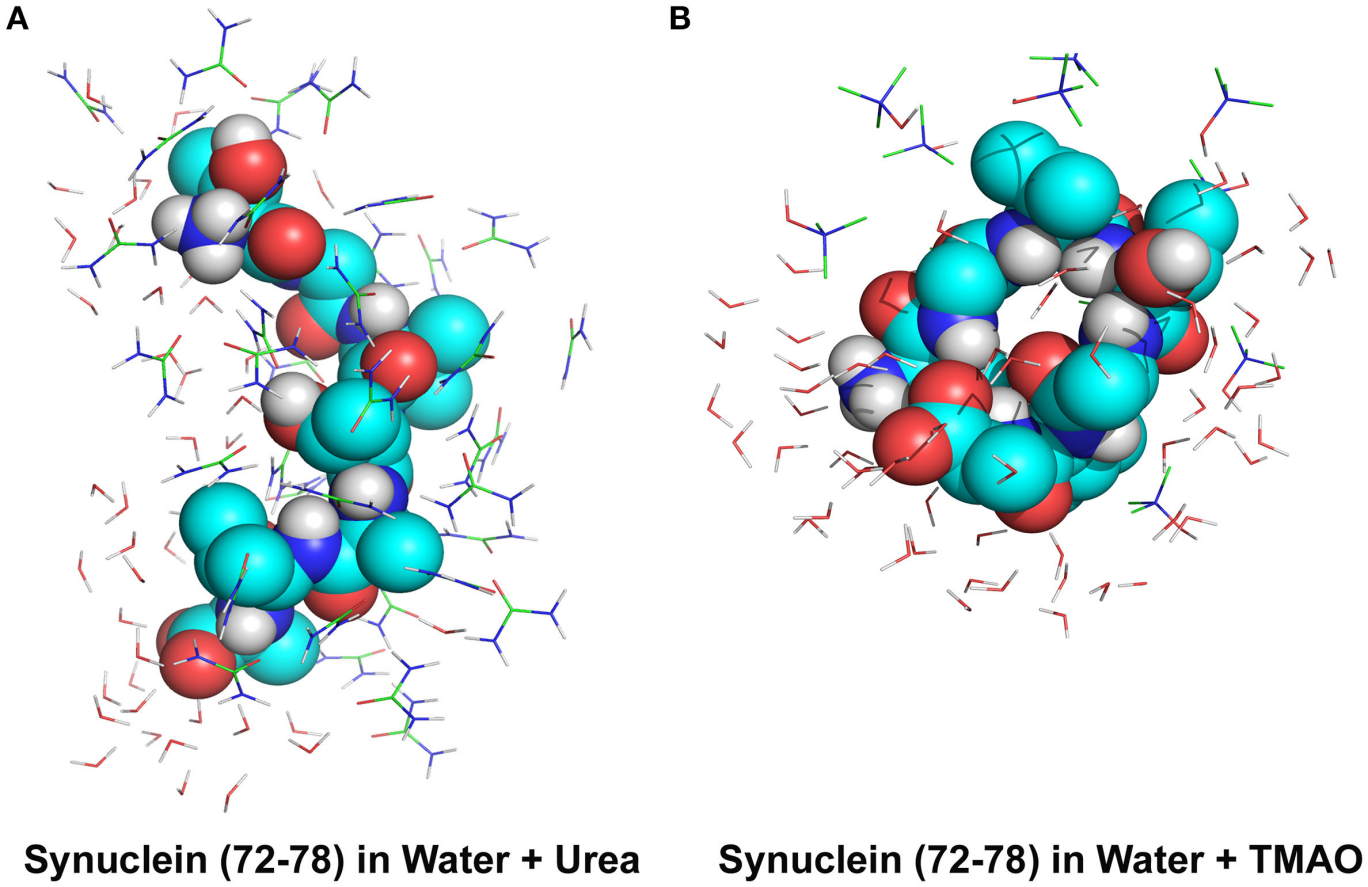
Peptide dehydration for systems containing **(A)** urea and **(B)** TMAO within the 5 Å of the Synuclein peptide surface. Major peptide dehydration was observed in case of urea while moderate peptide dehydration was observed for TMAO containing system. Urea molecules have a tendency to bind more closely to peptide compared to TMAO.

**Figure 5:**
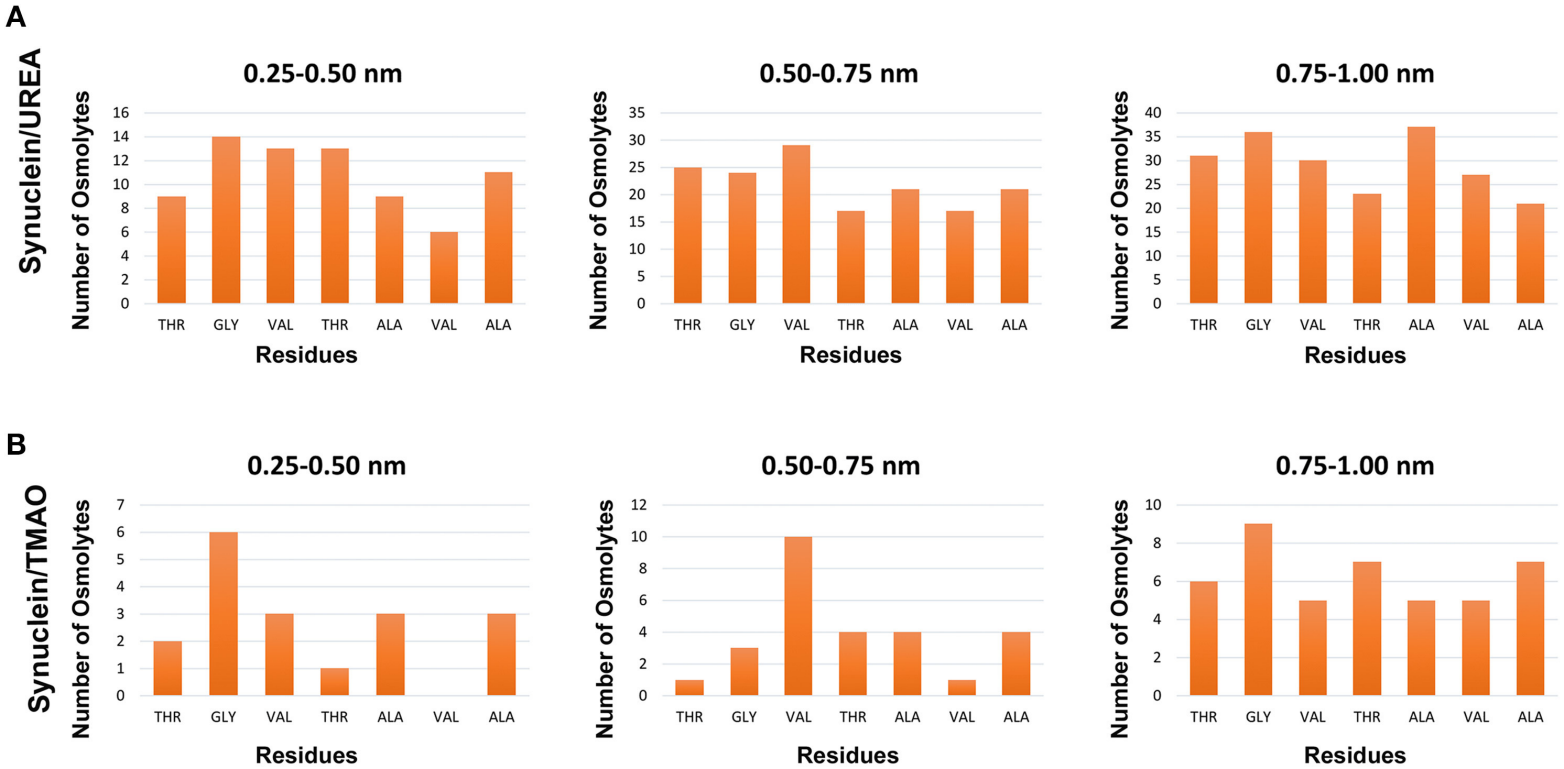
Distribution of osmolytes at various distances around the backbone of the Synuclein peptide monomer, **(A)** Synuclein (urea) and **(B)** Synuclein (TMAO). It can be observed urea crowded hydrophobic amino acids glycine, alanine, and valine resulting in significant reduction of water molecules from the surface of the peptide.

To explain the peptide dehydration for systems containing urea and TMAO, the amount of water molecules within 5 Å of the α-synuclein peptide surface was calculated. Tables 2, 3 show the number of water and osmolytes found within 5 Å of the surface of whole peptide as well as for each residue of the peptide for all the three systems, Synuclein_water_, Synuclein_Urea+water_, and Synuclein_TMAO+water_, respectively. A significant decrease in the number of water molecules in case of urea was observed which was hydrated by only 44 water molecules, as compared to 175 water molecules in case of Synuclein_water_. This decrease in the number of water molecules can be attributed to the mechanism that the urea molecules preferentially bound more to the side chains and backbone of peptide, consequently leading to exclusion of water from the peptide's surface. A reduction in the number of water molecules from 175 to 57 was also observed in case of Synuclein_TMAO+water_, though to a lesser extent as compared to urea. This shows that lesser number of TMAO molecules (only 10) was bound to the surface of peptide resulting in water-to-TMAO ratio of 5.70 in comparison to urea in which 36 molecules were very closely bound to the peptide resulting in water-to-urea ratio of 1.22. The results obtained demonstrate that urea prefers enhanced interaction with the peptide more as compared to TMAO. The dehydration patterns of the peptide in urea and TMAO containing systems is clearly visible from Figure 4 which depicts prominent peptide dehydration in case of urea, while only moderate peptide dehydration for TMAO containing system was observed. A similar depletion of water from the peptide surface was observed by Sarma and Paul (2013) in case of S-peptide analog (Sarma and Paul, 2013). A distribution of osmolytes at various distances around the backbone of the α-synuclein peptide monomer has been shown in Figure 5. As is evident from the figure, urea tends to cluster more around the hydrophobic amino acids, glycine, alanine, and valine which explains the significant reduction of water molecules from the peptide surface in case of systems containing urea. TMAO also crowded hydrophobic amino acids glycine, alanine and valine but to a lesser extent in comparison to urea.

**Table 2:** Shows the number of water and osmolytes found within 5 Å of the surface of peptide for all the three systems, Synuclein_water_, Synuclein_Urea+water_, and Synuclein_TMAO+water_.

**Solvent + Osmolytes**	**No. of waters**	**No. of osmolytes**	**Water/Osmolytes**
Water	175	0	N/A
Water + Urea	44	36	1.22
Water + TMAO	57	10	5.70

**Table 3:** Shows the number of water and osmolytes found within 5 Å of each residue of the peptide for all the three systems, Synuclein_water_, Synuclein_Urea+water_, and Synuclein_TMAO+water_.

**Residue**	**No. of waters**	**No. of osmolytes**		**Water/Osmolytes**	
		**Water + Urea**	**Water + TMAO**	**Water/Urea**	**Water/TMAO**
THR	23	10	1	2.30	23
GLY	13	9	2	1.14	6.50
VAL	10	14	6	0.71	1.67
THR	10	14	4	0.71	2.50
ALA	18	14	1	1.29	18
VAL	24	9	3	2.67	8
ALA	35	6	0	5.83	N/A

### Interaction mechanisms of osmolytes around peptide surface

As is reflected from Figure 4, urea molecules tend to preferentially interact more among themselves which can be due to similar chemical formula of urea, i.e., CO and NH bond, to the peptide backbone. It was also observed that urea molecules showed favored interaction more closely to peptide since the peptide backbone formed more energetically favorable bonds with the nearby urea molecule instead of finding a corresponding amino acid residue of the peptide (Canchi and García, 2013). This can also be correlated to a reduced number of intramolecular hydrogen bonds in case of urea which subsequently lead to the formation of unfolded and extended conformations of the peptide. However, the mechanism of interactions in case of TMAO was entirely different from urea. We observed that TMAO molecules did not interact much with the nearby TMAO molecules and rather preferred interaction with the adjacent water molecules. The lesser number of TMAO molecules binding to the peptide may be due to the entropically unfavorable interactions with the amide NH of the peptide backbone as reported by Cho et al. (2011).

### Role of hydrogen bonds and salt bridges in transition of conformational ensembles

Various studies have reported that the intrinsically disordered peptides show a very different theoretical behavior than the well-structured folded proteins. Müller-Späth et al. (2010) reported that the polyampholyte theory very well explains the influence of charged amino acids on the dimensions of the protein chain and thus can be applied for predicting the conformations in case of IDPs and unfolded proteins (Müller-Späth et al., 2010). In another study, Weinkam et al. (2009) carried out the simulation of denatured ensembles of cytochrome c and showed that the hydrophobic and electrostatic interactions lead to variations in conformations of different regions of the sequences in case of IDP (Weinkam et al., 2009). Here we have attempted to explore the conformations of intrinsically disordered α-synuclein in the presence of osmolytes which cause a population shift in the conformational ensembles of the peptide. It was seen that the extended conformations of the peptide appeared when the probability of the occurrence of stable intramolecular hydrogen bonds was less. This may be due to the reason that peptides can easily change their conformations without having any impact on salt bridges as these are entropically favorable transitions in absence of hydrogen bonds. Thus, the transitions between the formations of compact and extended forms of peptide can be seen as interplay of presence or absence of hydrogen bonds and possibilities of occurrence of salt bridges. As in present study, we observed that urea promoted the formation of stable extended forms of the peptide and was found to be preferentially closely interacting with the peptide whereas TMAO resulted in stabilized compact conformations forming more hydrogen bonds. Thus, it can be concluded that breakage and formation of intramolecular hydrogen bonds and salt bridges play a remarkable role in the formation of different conformational ensembles in case of intrinsically disordered peptides.

## Discussion

IDP have been known to be involved in many diseases which include various neurodegenerative disorders, cardiovascular diseases and cancer. Exploring the structure and functions of these disease associated proteins could be of great advantage for drug development for countering the diseases. Alpha-synuclein is one such IDP, the aggregation and misfolding of which has been the cause of many synucleinopathies and toxicity leading to various diseases. The human α-synuclein is a neuronal protein which is intrinsically disordered and is a major component of Lewy bodies which are aggregates of proteins that contribute to physiology of PD and various other neurodegenerative diseases involving protein aggregation. It has been reported in various studies that the changes in the conformation of monomeric α-synuclein lead to its aggregation in PD-inflicted brains and thus characterizing structure of α-synuclein is highly important. Unfortunately, IDPs are highly dynamic in nature and undergo a plethora of conformational rearrangements which makes the use of experimental approaches for designing novel drugs very limited. Computational methods such as REMD during which n-independent replicas are generated and simulated concurrently at a range of temperatures, have been successfully used to investigate the folding/misfolding of various IDPs.

The monomeric conformation of α-synuclein protein has been described to have characteristics of IDPs. The physiological function of alpha-synuclein has been observed to be acting as molecular chaperones promoting the formation of large protein complexes (Burré et al., 2010), vesicle trafficking and release of neurotransmitters (Norris et al., 2004). However, a conformational change in monomeric alpha-synuclein has been reported to lead to its aggregation into fibrils playing an important role in the pathology of PD (Spillantini et al., 1997; Moore et al., 2005). Understanding the conformational changes in monomeric form of alpha-synuclein is thus essential to prevent its aggregation and thus PD. Osmolytes belong to the family of small-molecule chaperones that play an important role in maintaining the folding and unfolding events of proteins. Urea shows a strong denaturing effect on proteins even at physiologically significant concentrations, however it has been put forward that the protecting osmolytes like TMAO can counter the denaturing effects of urea (Yancey and Somero, 1979). Here, we carried out REMD simulations on α-synuclein peptide in the presence of osmolytes urea acting as denaturant and TMAO acting as protectant osmolyte. A remarkably distinct impact of the osmolytes on the conformational behavior of the peptide was observed. In case of urea, the peptide was found having an extended form whereas a population shift into compact and folded conformations was seen in case of TMAO. In the present study we observed that the Parkinson's disease linked α-synuclein which is an intrinsically disordered protein adopted compact and extended conformations in the presence of protectant, TMAO, and denaturant, urea, osmolytes, respectively. The changes in local structure of the protein, α-synuclein, could further potentiate altered binding or folding ultimately resulting in modified functions. The effects of urea and TMAO on the structures of alpha-synuclein could probably prevent the alpha-synuclein aggregation and formation of insoluble fibrils leading to Parkinson's disease. Various studies have been carried out to study the conformational behavior of alpha-synuclein using REMD. A study was conducted on the monomeric form of the protein, synuclein, using single molecule experiments for the impact of the osmolytes (Ferreon et al., 2012). Their results demonstrated the ability of urea and TMAO to shift the conformations of alpha-synuclein between compact or extended structures. In another study, the effect of TMAO on unfolded α-synuclein was studied and observed that TMAO caused folding of the peptide in biphasic manner (Uversky et al., 2001). Mane and Stepanova (2016) investigated the folding dynamics of alpha-synuclein in initially unfolded form in water using an all-atom molecular dynamics simulations and essential dynamics (Mane and Stepanova, 2016). The synuclein constructs included a monomer, dimer and a tetramer. They observed that the initially unfolded form adopted globular conformation dominated by random coils and isolated transient β-bridges in case of monomeric form. The dimers were dominated by significant stable β-sheets which was not the case for tetramers which were less dependent on β-sheets for stability. In yet another study, the oligomerization of a fibril-forming α-synuclein peptide (residues 71–82), trimers and tetramers, was studied using REMD and it was observed that the conformational stability of the peptide increased as the size of oligomer increased from a dimer to a tetramer (Park et al., 2012).

A decrease in the number of hydrogen bonds was observed in Synuclein_Urea+water_ indicating the formation of extended structures of the peptide. Additionally, we studied the hydration patterns of the peptide and found that urea repels water from the peptide surface and instead itself preferentially binds numerously to the peptide backbone and side chains. On the other hand TMAO itself does not interact much with the peptide and preferentially distributes water around the peptide surface resulting in stabilized compact peptide conformations. This study reports the detailed role of external agents, i.e., osmolytes urea and TMAO, on the conformational behavior of intrinsically disordered α-synuclein peptide. The results obtained in the present study could provide a better understanding of the structural behavior and aggregation patterns of PD linked α-synuclein.

## Author contributions

SJ, AK, and AG: conceived and designed the experiments; SJ and AK: performed the analysis; SJ, AK, AS, SG, and AG: analyzed the data; AG: contributed reagents/materials/analysis tools; All authors contributed to the writing of the manuscript.

### Conflict of interest statement

The authors declare that the research was conducted in the absence of any commercial or financial relationships that could be construed as a potential conflict of interest.

## Supplementary material

Figure S1 Population densities of Synuclein peptide conformations at T = 311.24K. **(A)** Synuclein (water), **(B)** Synuclein (urea), and **(C)** Synuclein (TMAO).

Figure S2 Population densities of Synuclein peptide conformations at T = 322.18K. **(A)** Synuclein (water), **(B)** Synuclein (urea), and **(C)** Synuclein (TMAO).

Figure S3 Population densities of Synuclein peptide conformations at T = 333.42K. **(A)** Synuclein (water), **(B)** Synuclein (urea), and **(C)** Synuclein (TMAO).

Figure S4 Population densities of Synuclein peptide conformations at T = 342.08K. **(A)** Synuclein (water), **(B)** Synuclein (urea), and **(C)** Synuclein (TMAO).

Figure S5 Population densities of Synuclein peptide conformations at T = 347.94K. **(A)** Synuclein (water), **(B)** Synuclein (urea), and **(C)** Synuclein (TMAO).
