# Supplementary figures and images for: Conformational Ensembles of α-Synuclein Derived Peptide with Different Osmolytes from Temperature Replica Exchange Sampling
Source: Front Neurosci. 2017 Dec 7;11:684. doi: 10.3389/fnins.2017.00684 (PMC5725442; doi:10.3389/fnins.2017.00684)

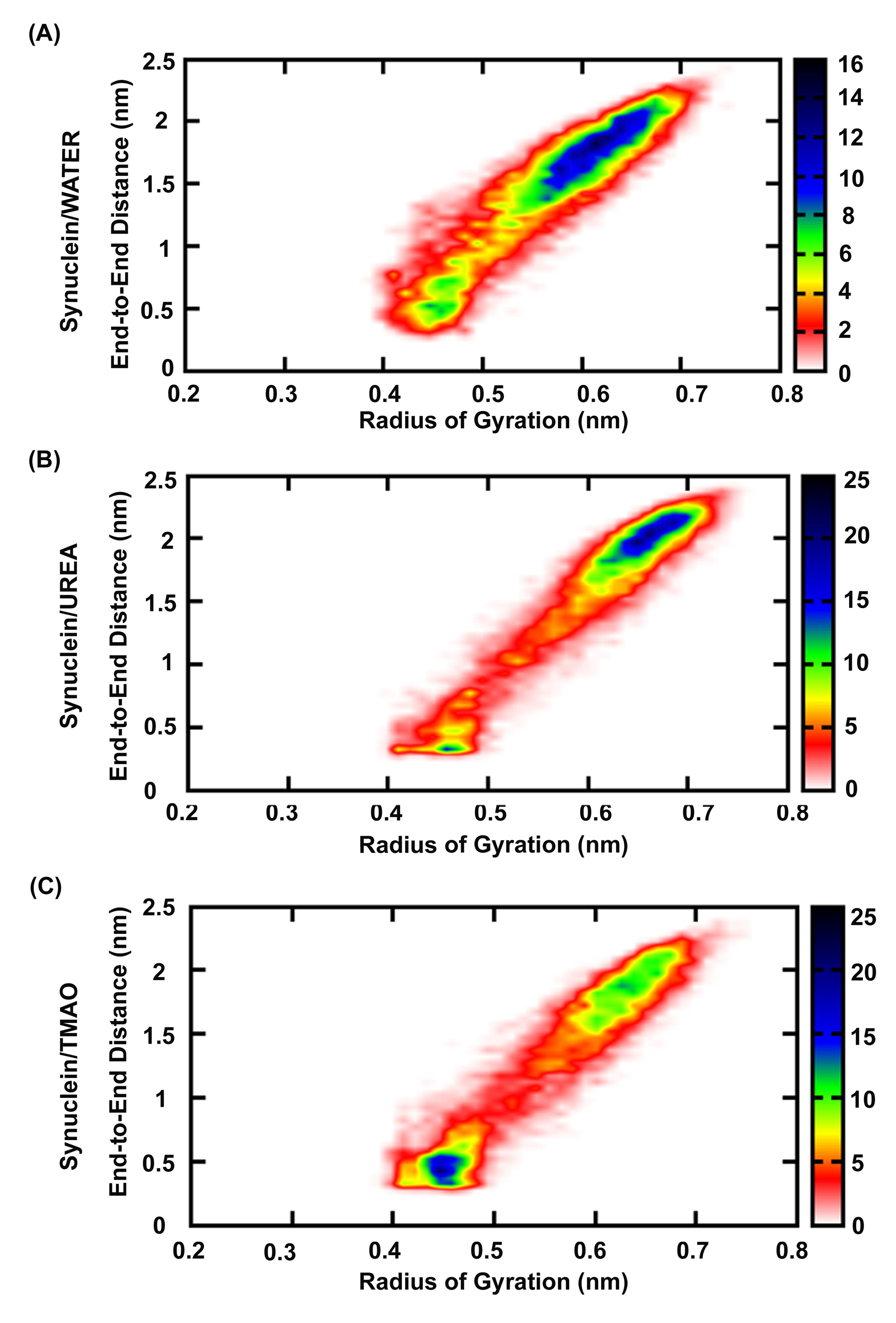

Supplement: Figure S1 — Population densities of Synuclein peptide conformations at T = 311.24K. (A) Synuclein (water), (B) Synuclein (urea), and (C) Synuclein (TMAO). [file Image1.TIF]

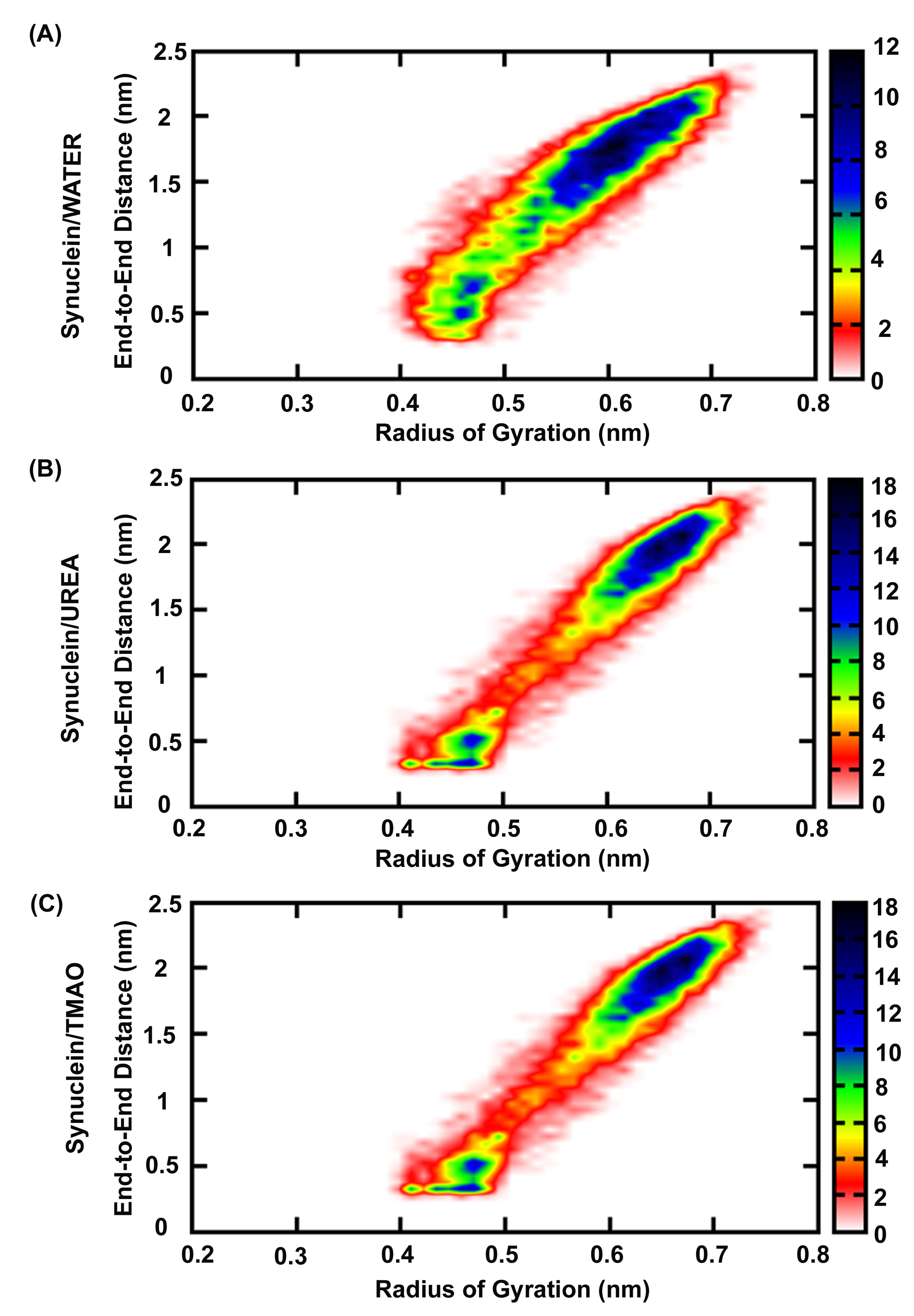

Supplement: Figure S2 — Population densities of Synuclein peptide conformations at T = 322.18K. (A) Synuclein (water), (B) Synuclein (urea), and (C) Synuclein (TMAO). [file Image2.TIF]

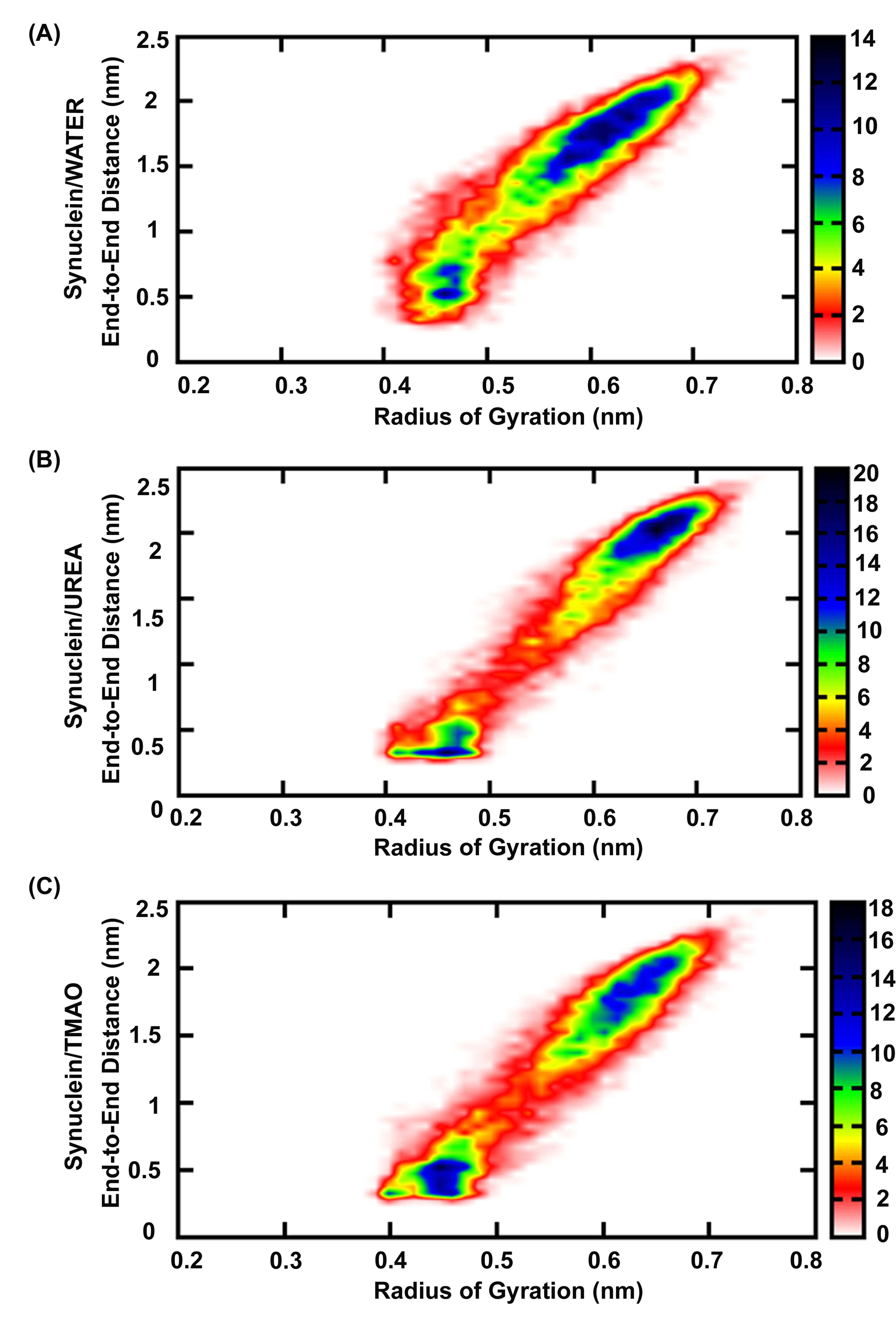

Supplement: Figure S3 — Population densities of Synuclein peptide conformations at T = 333.42K. (A) Synuclein (water), (B) Synuclein (urea), and (C) Synuclein (TMAO). [file Image3.TIF]

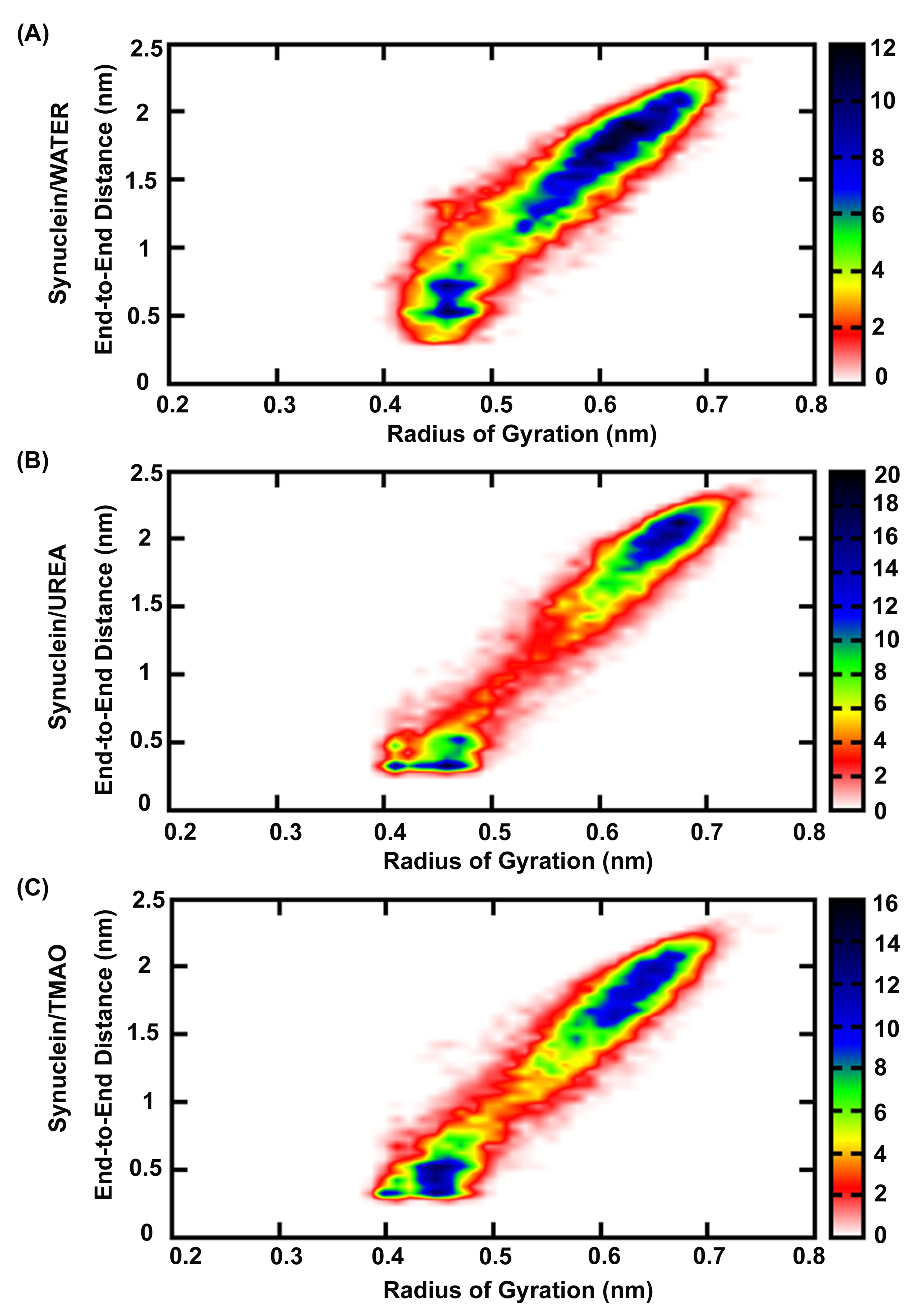

Supplement: Figure S4 — Population densities of Synuclein peptide conformations at T = 342.08K. (A) Synuclein (water), (B) Synuclein (urea), and (C) Synuclein (TMAO). [file Image4.TIF]

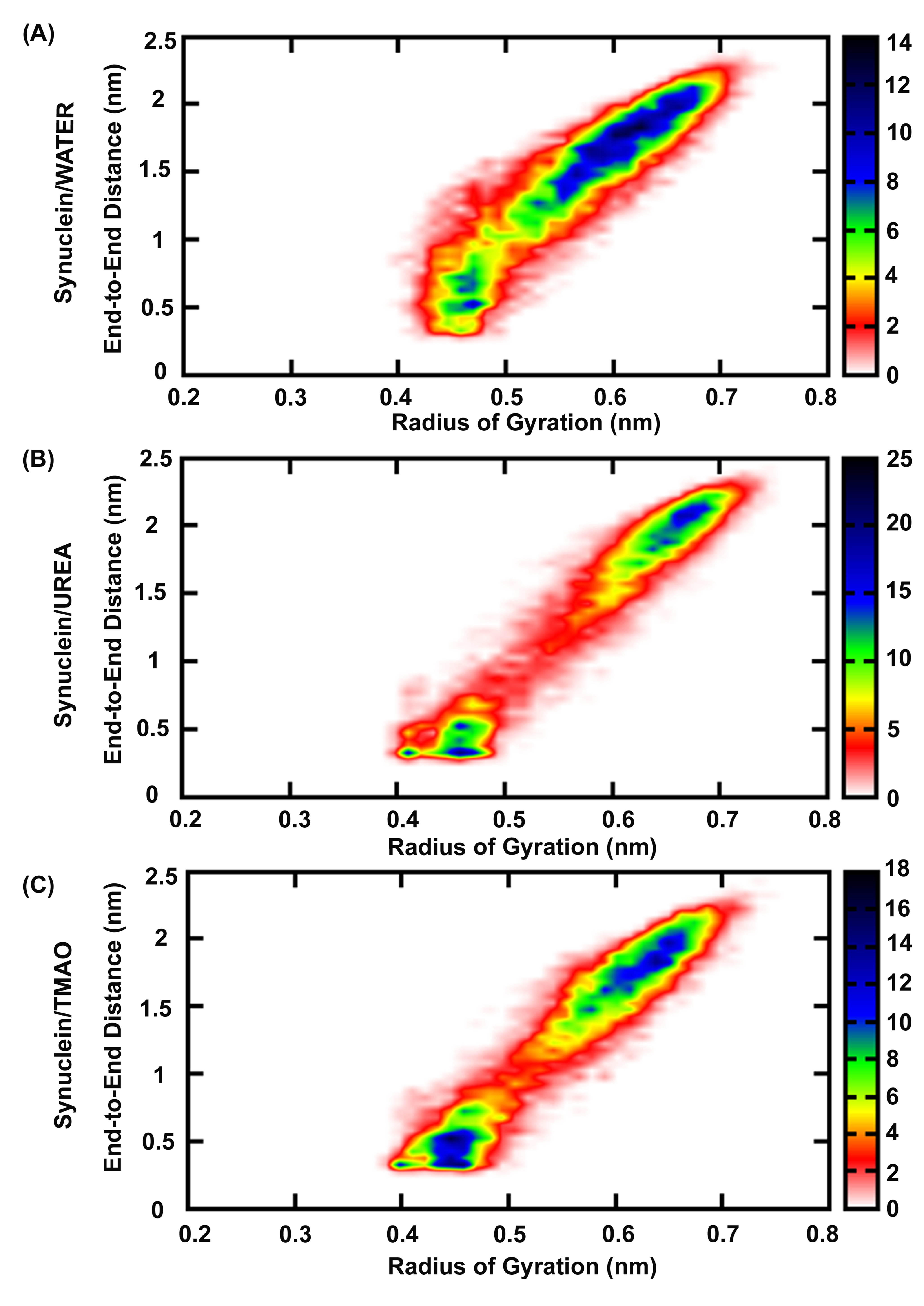

Supplement: Figure S5 — Population densities of Synuclein peptide conformations at T = 347.94K. (A) Synuclein (water), (B) Synuclein (urea), and (C) Synuclein (TMAO). [file Image5.TIF]
