# Supplementary material for: Conformational Ensembles of α-Synuclein Derived Peptide with Different Osmolytes from Temperature Replica Exchange Sampling
Source: Front Neurosci. 2017 Dec 7;11:684. doi: 10.3389/fnins.2017.00684 (PMC5725442; doi:10.3389/fnins.2017.00684)
Supplement: Supplementary file 6 [file Table1.DOCX]

| **Temperatures (K)** | **Synuclein peptide in respective solvent/s** | **Average Rₑₑ** | **Average R_g_** |
| --- | --- | --- | --- |
| 300.61 | Synuclein_water_ | 1.39 nm | 0.55 nm |
|  | Synuclein_Urea + water_ | 1.53 nm | 0.59 nm |
|  | Synuclein_TMAO + water_ | 1.27 nm | 0.54 nm |
| 311.24 | Synuclein_water_ | 1.40 nm | 0.56 nm |
|  | Synuclein_Urea + water_ | 1.50 nm | 0.59 nm |
|  | Synuclein_TMAO + water_ | 1.26 nm | 0.55 nm |
| 322.18 | Synuclein_water_ | 1.39 nm | 0.56 nm |
|  | Synuclein_Urea + water_ | 1.45 nm | 0.58 nm |
|  | Synuclein_TMAO + water_ | 1.24 nm | 0.55 nm |
| 333.42 | Synuclein_water_ | 1.37 nm | 0.55 nm |
|  | Synuclein_Urea + water_ | 1.40 nm | 0.58 nm |
|  | Synuclein_TMAO + water_ | 1.27 nm | 0.55 nm |
| 342.08 | Synuclein_water_ | 1.36 nm | 0.55 nm |
|  | Synuclein_Urea + water_ | 1.39 nm | 0.57 nm |
|  | Synuclein_TMAO + water_ | 1.28 nm | 0.55 nm |
| 347.94 | Synuclein_water_ | 1.38 nm | 0.56 nm |
|  | Synuclein_Urea + water_ | 1.36 nm | 0.57 nm |
|  | Synuclein_TMAO + water_ | 1.27 nm | 0.55 nm |

**Table S1** Average R_ee_ i.e. peptide end-to-end distance (C to N terminal) and R_g_ i.e. radius of gyration of Synuclein monomer at various temperatures.
